# Exploring Heterogeneity in Meta‐Analysis Using Trial‐Level Characteristics: A Tutorial

**DOI:** 10.1002/cesm.70099

**Published:** 2026-08-03

**Authors:** Peter J. Godolphin, Conor D. Tweed, David J. Fisher

**Affiliations:** ^1^ UCL Innovative Clinical Trials Unit, Institute of Clinical Trials and Methodology University College London London UK

## Abstract

This tutorial describes how to explore across‐trial heterogeneity in meta‐analysis using trial‐level characteristics. It describes how to carry out a subgroup analysis for a trial‐level characteristic with more than two categories and provides approaches for analysing continuous trial‐level characteristics. The tutorial introduces meta‐regression, outlines its intended applications, and explains how to interpret meta‐regression results. Common pitfalls when undertaking trial‐level subgroup analysis and meta‐regression are also discussed. A complementary micro‐learning module is provided to support additional learning of these concepts.

## 1 Introduction

A previous tutorial [1] introduced the concept of subgroup analysis and outlined how it can be used to investigate and explain across‐trial heterogeneity by using trial‐level characteristics. It also described the how to carry out a subgroup analysis within a meta‐analysis when a trial‐level characteristic has two categories. The present tutorial extends this to situations where trial‐level characteristics have more than two categories and those that are measured on a continuous scale, highlighting key methodological considerations to avoid common errors.

## 2 Trial‐Level Subgroup Analysis With More Than Two Categories

**Figure 1:**
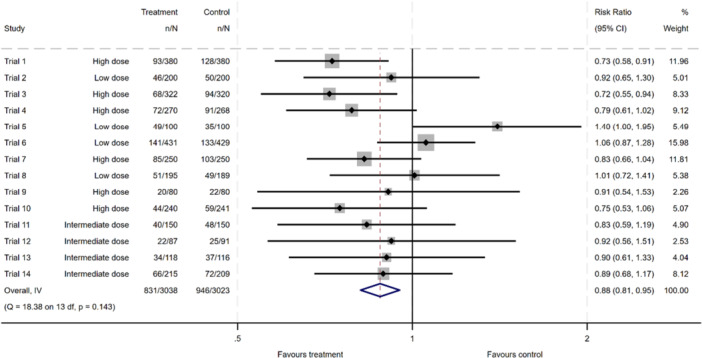
Updated hypothetical hypertension meta‐analysis for an outcome of mortality.

In the previous tutorial [1], a hypothetical meta‐analysis was considered in which 10 hypertension trials of a blood‐pressure lowering drug with a binary outcome of mortality were grouped by dose (Low vs. High). Suppose now that four additional hypertension trials were identified, each classified as using an Intermediate dose (Trials 11–14). Figure 1 presents the forest plot from an inverse variance common‐effect meta‐analysis of all 14 trials. Visual inspection of the forest plot indicates potential inconsistency (or “heterogeneity”) across the trials.

**Figure 2:**
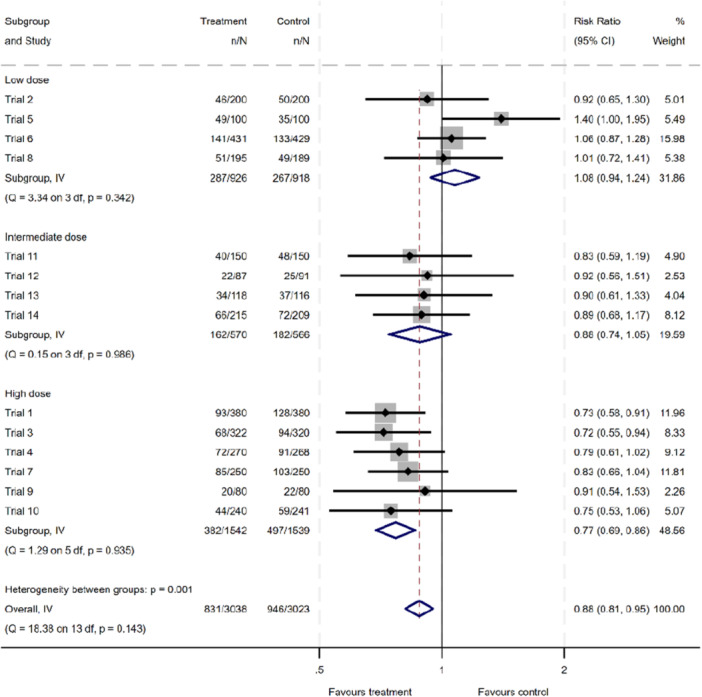
Trial‐level subgroup analysis by dose (three dose categories).

The approach introduced in the previous tutorial [1] for two groups can be extended in a straightforward manner to allow for three groups. When trials are partitioned into three subsets by dose, the calculations for the Low‐dose and High‐dose remain unchanged, and the heterogeneity Q statistic is additionally computed for the Intermediate‐dose subgroup (Figure 2).

From Figure 2, the heterogeneity (Cochran's Q) statistics for all three dose subgroups are non‐significant. This suggests that the observed heterogeneity may be at least partially explained by dose. A formal test for subgroup differences can be carried out by simply extending the approach described previously [1]. The total amount of heterogeneity ($Q_{T}$) is split into heterogeneity identified within ($Q_{W}$) and between ($Q_{B}$) the subgroups. For a trial‐level subgroup analysis with S subgroups this is expressed as:

$$Q_{T}=Q_{B}+Q_{W},\;\text{where }Q_{W}=\sum_{i=1}^{S}Q_{i},$$

From Figure 2, $Q_{W}=3.34+0.15+1.29=4.63$, and $Q_{T}=18.38$. Therefore, $Q_{B}=18.38-4.78=13.60$, and comparing this value to a *χ* ^2^ distribution with two degrees of freedom (as S−1=3−1=2) gives a *p* value of 0.0011 for the test of subgroup differences.

A similar process would be followed for a trial‐level characteristic with four or more groups. It should be noted that a given subgrouping may not provide the optimal explanation of heterogeneity. Therefore, subgroups explored should be justified on the basis of clinical reasoning and data availability.

## 3 Handling Continuous Trial‐Level Characteristics

In some settings, dose might be measured on a continuous scale, for example ranging from 100 to 1000 mg across trials. One way of analysing such a characteristic would be to categorize the continuous variable (e.g., Low = 100 to 300 mg; Intermediate = 301 to 700 mg; High = 701 to 1000 mg), so that trial‐level subgroup analysis can be applied as described above. Such categorization might sometimes aid interpretation and better support clinical decision making, for example if dose categories are commonly recognized and used across the world. However, categorization does not make efficient use of information and reduces statistical power. Meta‐regression is an alternative approach, which makes full use of the continuous range of dose values.

## 4 Meta‐Regression: How and When to Use It

Meta‐regression is a generalization of trial‐level subgroup analysis that enables continuous as well as categorical characteristics to be explored simultaneously. In meta‐regression, the outcome of interest is the effect estimate used in the meta‐analysis, and the independent variables are the trial characteristics that are hypothesized to explain heterogeneity (e.g., dose in the preceding example). For outcome Y and a set of n trial characteristics $X_{1},X_{2},\ldots ,X_{n}$, we have, for each trial i:

$$Y_{i}=\alpha +\beta_{1}X_{1i}+\beta_{2}X_{2i}+\ldots +\beta_{n}X_{\mathrm{ni}}.$$

Each coefficient $\beta_{1},\beta_{2},\ldots ,\beta_{n}$ represents the predicted change in outcome for a one‐unit change in the associated characteristic. This can be illustrated using the earlier examples. For a binary characteristic, the value of X is either zero or one, and the value of coefficient β represents the change in Y as X changes from zero to one: that is, a comparison of e.g. High with Low dose. A three‐category characteristic is represented using two dummy variables $X_{1}\;\text{and}\;X_{2}$. The coefficient $\beta_{1}$ can be interpreted as the comparison of, say, Intermediate with Low dose; and $\beta_{2}$ as the comparison of High with Low dose. Finally, if the dose characteristic is continuous, then the value of coefficient β quantifies the change in Y as a result of increasing the dose by a single unit.

Meta‐regression can be performed under a common‐effect, fixed‐effects or random‐effects model [2]. However random‐effects models are generally preferred in order to protect against the risk of false‐positive findings [3]. Meta‐regression might be preferred to simpler subgroup analysis if there are multiple candidates for explaining the observed heterogeneity, and/or if continuous data is available for one or more characteristics. As a type of regression model, it may also be generalized in various ways, such as multilevel meta‐regression [4]. Furthermore, if the covariates are each transformed to have zero mean, then the intercept α is the predicted overall effect size at the average value of the covariates [5].

## 5 Key Tips When Exploring Heterogeneity Using Trial‐Level Characteristics

1. A substantial number of studies (> 10) is required before heterogeneity can be explored reliably using trial‐level characteristics. Subgroup analysis and meta‐regression is not advised with only a small number of included trials, or in cases where there are a very small number of studies in one subgroup (e.g., 1 Low dose trial, compared with 15 High dose trials).
2. Subgroup analyses and meta‐regression are not randomized evidence and should be interpreted accordingly. Do not compare effect estimates in different subgroups by considering the meta‐analysis results from each subgroup separately [6]. Instead, calculate a test for subgroup differences [1]. Consider assessing the credibility of any identified subgroup differences (e.g., using ICEMAN [7]).
3. As more subgroup analyses and meta‐regressions are performed, the likelihood of chance findings (false positives and false negatives) rapidly increases. It is important that only a small number of characteristics are explored, that each characteristic has a clear clinical or biological rationale for inclusion and, ideally, are pre‐specified in a review protocol.
4. Be aware that considering a characteristic that varies within a trial (i.e., participant‐level) as a trial‐level characteristic (e.g., mean age) could introduce aggregation bias [8].

## 6 FURTHER Reading and Online Content

More information on trial‐level subgroup analysis and meta‐regression can be found in Chapter 10.11 of the Cochrane Handbook for Systematic Reviews of Interventions [9] and a tutorial by Kanellopoulou et al. [6]. A linked earlier tutorial introduces the concept of subgroup analysis in a single trial and in meta‐analysis [1]. Cochrane Training has produced a micro‐learning module covering trial‐level subgroup analysis to accompany both tutorials.

## Author Contributions

**Peter J. Godolphin:** conceptualization, formal analysis, writing – original draft, writing – review and editing. **Conor D. Tweed:** conceptualization, writing – review and editing. **David J. Fisher:** conceptualization, writing – original draft, writing – review and editing.

## Conflicts of Interest

The authors declare no conflicts of interest.

## 1 Peer Review

The peer review history for this article is available at https://www.webofscience.com/api/gateway/wos/peer-review/10.1002/cesm.70099.

## Supporting information


Supporting File

## Data Availability

All data used in this article is aggregate data and is “hypothetical.” This can be extracted from Figure 1.
